# A short decision time for transcatheter embolization can better associate mortality in patients with pelvic fracture: a retrospective study

**DOI:** 10.3389/fmed.2023.1329167

**Published:** 2024-01-08

**Authors:** Yosuke Mizuno, Takahito Miyake, Hideshi Okada, Takuma Ishihara, Norihide Kanda, Masahiro Ichihashi, Ryo Kamidani, Tetsuya Fukuta, Takahiro Yoshida, Shoma Nagata, Hiroshi Kawada, Masayuki Matsuo, Shozo Yoshida, Shinji Ogura

**Affiliations:** ^1^Department of Emergency and Disaster Medicine, Gifu University Graduate School of Medicine, Gifu, Japan; ^2^Center for One Medicine Innovative Translational Research, Gifu University Institute for Advanced Study, Gifu, Japan; ^3^Innovative and Clinical Research Promotion Center, Gifu University, Gifu, Japan; ^4^Department of Radiology, Gifu University Graduate School of Medicine, Gifu, Japan; ^5^Abuse Prevention Center, Gifu University Graduate School of Medicine, Gifu, Japan

**Keywords:** transcatheter arterial embolization, pelvic injury, mortality, hemostasis strategies, retrospective study

## Abstract

**Background:**

Early use of hemostasis strategies, transcatheter arterial embolization (TAE) is critical in cases of pelvic injury because of the risk of hemorrhagic shock and other fatal injuries. We investigated the influence of delays in TAE administration on mortality.

**Methods:**

Patients admitted to the Advanced Critical Care Center at Gifu University with pelvic injury between January 2008 and December 2019, and who underwent acute TAE, were retrospectively enrolled. The time from when the doctor decided to administer TAE to the start of TAE (needling time) was defined as “decision-TAE time.”

**Results:**

We included 158 patients, of whom 23 patients died. The median decision-TAE time was 59.5 min. Kaplan–Meier curves for overall survival were compared between patients with decision-TAE time above and below the median cutoff value; survival was significantly better for patients with values below the median cutoff value (*p* = 0.020). Multivariable Cox proportional hazards regression analysis revealed that the longer the decision-TAE time, the higher the risk of mortality (*p* = 0.031). TAE duration modified the association between decision-TAE time and overall survival (*p* = 0.109), as shorter TAE duration (procedure time) was associated with the best survival rate (*p* for interaction = 0.109).

**Conclusion:**

Decision-TAE time may play a key role in establishing resuscitation procedures in patients with pelvic fracture, and efforts to shorten this time should be pursued.

## 1 Introduction

Pelvic injury is often associated with hemorrhagic shock and other fatal injuries (1). Hemorrhage-related mortality rate may be as high as 40%, and overall mortality rate in these patients may be 10–32%, even if hospitalized in a level 1 trauma center (2–5). In the emergency department (ED), the definitive treatments to achieve timely hemodynamic stabilization in patients with pelvic injury include transcatheter arterial embolization (TAE) and pre-peritoneal packing (PPP) (1, 6, 7); other treatments include arterial cross-clamping and resuscitative endovascular occlusion of the aorta (REBOA) (8, 9).

The literature contains many reports on the relative advantages of TAE and PPP (10–13); TAE, a less invasive procedure, has become widely accepted as a safe and efficacious substitute for direct surgical intervention (10). Conversely, considerable delays in performing embolization and a lack of readily available experts in angiography have been highlighted (10, 12). The mortality rates of patients treated with TAE range from 16 to 50% (14, 15), which is higher than that of patients treated with PPP (12).

Recent reports suggest that early administration of TAE results in low mortality rates; and the so-called “door-to-angioembolization time” should be shorter for better outcomes (4, 16). The true effectiveness of shortening the delay before administration of TAE can be confirmed using “decision-TAE time,” which represents the time from the decision to administer TAE to its actual administration.

In this study, we aimed to investigate how decision-TAE time influenced mortality in patients with pelvic trauma.

## 2 Materials and methods

### 2.1 Study design and ethics statement

This observational study used retrospectively collected data and adhered to the STrengthening the Reporting of OBservational Studies in Epidemiology (STROBE) statement. The study protocol is available. Ethics approval was obtained from the medical ethics committee of Gifu University Graduate School of Medicine, Gifu, Japan (Institutional Review Board approval No. 2020-061). The need for informed consent from the patients was waived by the medical ethics committee of the institution because of the study’s retrospective nature. This study adhered to the ethical guidelines for medical and health research involving humans, established by the Japanese government.

### 2.2 Study setting

Gifu University Hospital (Gifu-shi, Japan) is the only advanced critical care center in this region. The region includes catchment areas populated by approximately 2 million people. Patients with pelvic injury who underwent acute TAE were included, if they were admitted to Gifu University’s advanced critical care center between January 2008 and December 2019. The attending emergency physicians were responsible for the trauma survey and treatment of these patients in the ED. Emergency physicians and interventional radiologists were involved in the decision-making process. In our institution, interventional radiologists and the equipment required for TAE are available 24 h a day, 365 days a year.

### 2.3 Selection criteria

Patients who received TAE for pelvic fracture injury from trauma, including other injuries, were enrolled in this study. Patients with out-of-hospital cardiac arrest, without a response to resuscitation, with missing data on the time course of TAE, and those who underwent PPP were excluded. We identified the patients using the facility’s diagnosis codes: pelvic fracture, pelvic ring fracture, iliac fracture, pubic fracture, ischial fracture, sacral fracture, acetabular fracture, and hip fracture dislocation. All the data including demographic and biological data on admissions, treatment process, and outcomes were collected from medical records.

### 2.4 Treatment

**Figure 1:**
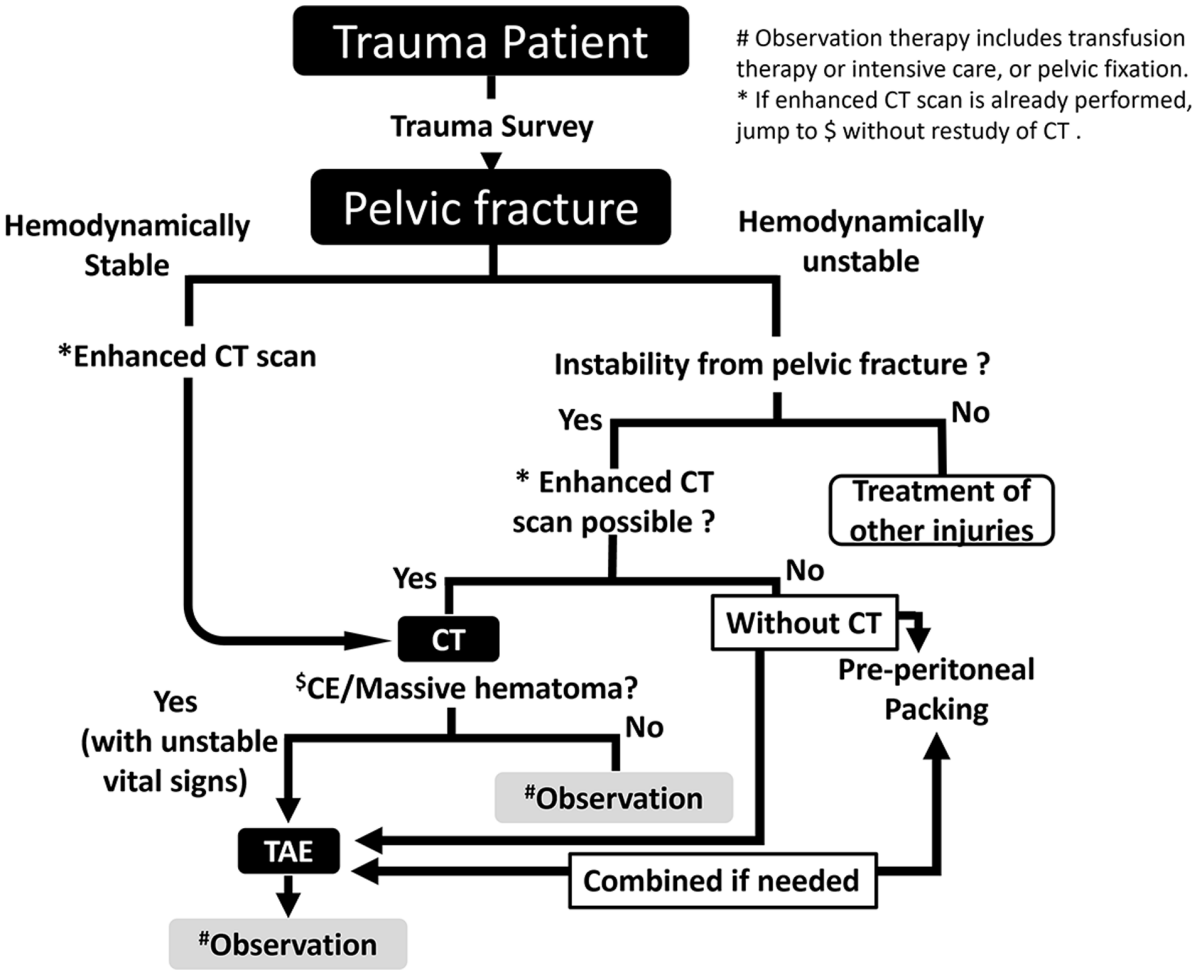
Treatment algorithm for pelvic injury. CT, computed tomography; TAE, transarterial catheter embolization.

At the advanced critical care center at Gifu University Hospital, we established a treatment algorithm based on the Eastern Association for the Surgery of Trauma recommendations (6). Patients who could not undergo computed tomography (CT) scan owing to hemodynamic instability were directly sent to undergo TAE. Some of them could not be prepared for TAE because of the risk of death or because PPP had just been performed; hence, TAE was added if needed. Other patients underwent a CT scan, and immediate TAE was initiated if necessary. If transferred patients had already undergone a CT and there was enough information to make a decision, additional examinations were bypassed and the patients were directly sent to undergo TAE. They were treated according to the algorithm shown in Figure 1. In some cases, REBOA was utilized, based on emergency physicians’ deci**s**ions. All patients needed TAE for hemostasis.

### 2.5 Definition of parameters

**Figure 2:**
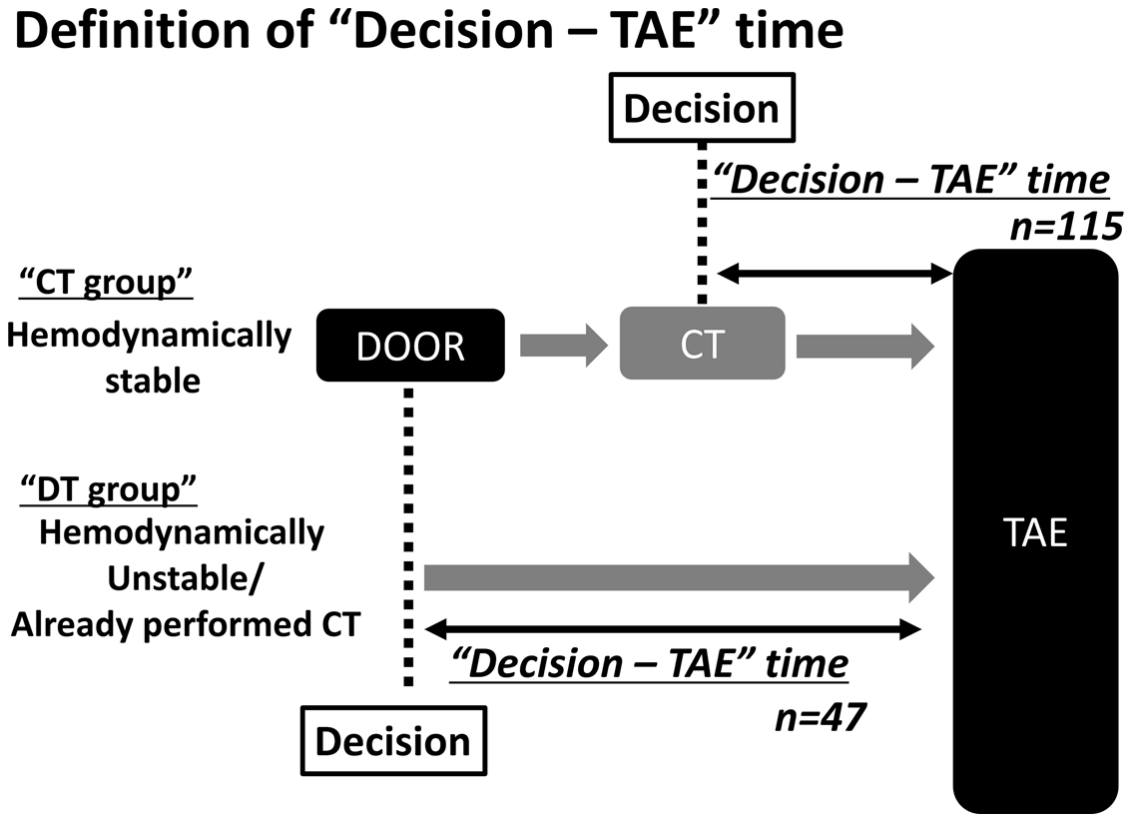
Definition of “Decision-TAE” time In the CT (CT-TAE) group, the decision-TAE time is defined as the time from the start of CT to the administration of TAE. In the door-TAE group, the decision-TAE time is defined as the time from arrival to administration of TAE. TAE, transarterial catheter embolization; CT, computed tomography.

Emergency physicians decided to administer TAE when: (1) the CT scan indicated massive hemorrhage from pelvic injury, or (2) the patient was hemodynamically unstable and did not undergo a CT scan or was transferred from another hospital after a CT scan. When the CT scan indicated massive hemorrhage, the decision-TAE time was defined as the time from starting the CT to TAE (CT-TAE group: CT group). When the patient did not undergo a CT scan or was transferred after a CT scan, the decision-TAE time was defined as the time from arrival at the ED to the administration of TAE (door-TAE group: DT group) (Figure 2).

Demographic and biological data on admission were collected from medical records. The injury severity score and the abbreviated injury score by body area (head, chest, abdomen, pelvis, and extremities) were calculated for each patient, and defined as “severe” if they scored ≥3 points.

### 2.6 Outcomes

The primary outcome of this study was the time from the end of TAE to death. There were 10 secondary outcomes, including parameters associated with TAE (decision-TAE time, number of arteries involved in TAE, localizations, embolic materials, TAE duration time, and number of secondary TAEs), treatment with REBOA, surgical management for pelvic fractures, length of hospital stay, and causes of death.

### 2.7 Statistical analysis

The baseline characteristics of the patients and the continuous variables were expressed as median and interquartile range (IQR) and categorical variables as counts and percentages. The sample size was calculated according to feasibility and not power, to avoid overfitting of the statistical model (17). For the primary analysis, Cox proportional hazards regression analysis was performed to confirm the effect of decision-TAE time on the time from the end of TAE administration to death. As age and sex are important characteristics affecting mortality (18, 19), the Cox proportional hazards model was adjusted for them to avoid confounding by patient baseline characteristics (18, 19). GCS and transfer were also incorporated into the model as covariates based on the previous report (20) and background of the study, as they are strongly related to outcome and decision-TAE time. Sensitivity analysis with GCS replaced by ISS or RTS was performed to confirm the robustness of the Hazard ratio for decision-TAE time. A sensitivity analysis of another perspective was also performed using a model with CT/DT group added as a covariate (Table 1).

**Table 1:** Multivariable Cox proportional hazards regression model.

Analysis	Factors	HR	95% LCI	95% UCI	*p* value
Primary analysis	Decision-TAE time	1.009	1.001	1.016	0.031
	GCS	0.812	0.735	0.898	<0.001
	Transfer	0.803	0.308	2.095	0.654
	Age	1.003	0.980	1.026	0.800
	Sex: female	0.339	0.116	0.991	<0.001
Sensitivity analysis 1	Decision-TAE time	1.009	1.001	1.018	0.025
	GCS	0.815	0.738	0.900	<0.001
	Transfer	0.618	0.189	2.022	0.426
	Age	1.002	0.979	1.025	0.037
	Sex: female	0.289	0.090	0.925	<0.001
	DT group	1.722	0.443	6.703	0.433
Sensitivity analysis 2	Decision-TAE time	1.007	0.999	1.015	0.073
	ISS	1.080	1.034	1.128	0.001
	Transfer	0.559	0.197	1.582	0.273
	Age	1.020	0.993	1.047	0.017
	Sex: female	0.260	0.086	0.784	0.001
	DT group	1.670	0.482	5.786	0.419
Sensitivity analysis 3	Decision-TAE time	1.011	1.003	1.019	0.010
	RTS	0.738	0.617	0.883	0.001
	Transfer	0.431	0.134	1.389	0.158
	Age	1.007	0.982	1.033	0.010
	Sex: female	0.230	0.076	0.700	0.002
	DT group	1.539	0.394	6.013	0.535

Multivariable Cox regression: TAE duration time				
Factors	HR	95% LCI	95% UCI	*p* value
TAE duration time	0.889	0.482	1.639	0.707
Age	1.007	0.984	1.032	0.543
Sex: female	0.365	0.135	0.989	0.048

Multivariable Cox regression: decision-TAE time. HR, hazard ratio; CI, confidence interval; UCI, upper CI; LCI, lower CI; TAE, transarterial catheter embolization; GCS, Glasgow coma scale.

To avoid overfitting, the number of covariates was limited to two or three (21). Therefore, if the calculated optimism parameter was <0.2, the model was not considered overfitting, even with the above four variables as covariates. The optimism (22) was estimated using 150 bootstrap resamples. Optimism assesses the magnitude of overfitting of regression model (a value less than 0.2 is considered as good) and was calculated using C-statistics by bootstrap samples. Subgroup analysis by DT group and CT group were performed using univariate Cox regression models (Supplementary Table S1). Kaplan–Meier estimation calculated the cumulative survival rate for each group divided by the median of the decision-TAE time. The difference in the cumulative survival rate between the two groups was confirmed using the log-rank test.

Similar to the model used for the primary analysis, the effects of the end of TAE administration on death were analyzed. In this secondary analysis, GCS score and transfer were not included as covariates because they were not related to the end of TAE administration. Additionally, an interaction term (decision-TAE time * TAE duration) was incorporated into the Cox proportional hazards model to test whether the effect of decision-TAE time on mortality was modified by including TAE duration. The hazard ratio for a unit increase in decision-TAE time or TAE duration with a 95% confidence interval was reported in each Cox proportional hazards analysis. A sensitivity analysis was performed using the Fine–Gray subdistribution hazard model, treating death from head trauma as a competing risk. Parameters that could influence the decision-TAE time on arrival were summarized for each group by dividing decision-TAE time into quartiles, and comparisons between groups were conducted using a Fisher’s exact test for categorical variables and a Kruskal–Wallis test for continuous variables. Imputation was not used for missing data because no data were missing for the primary outcome. A value of *p* (two-sided) <0.05 was considered significant. No adjustment was made for multiple comparisons because all analyses were exploratory. All statistical analyses were performed using the R version 4.2.2.^1^

## 3 Results

### 3.1 Patient demographics

**Figure 3:**
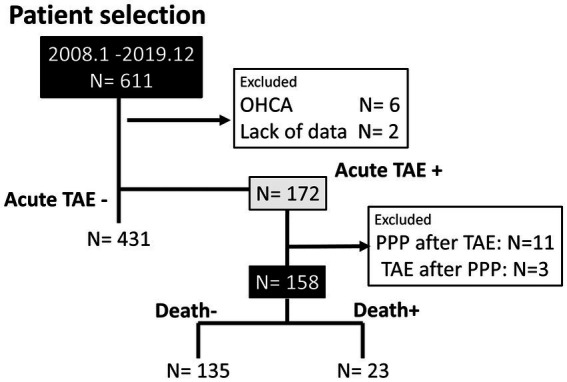
Patient selection. Flowchart diagram of eligible and excluded patients. OHCA, out-of-hospital cardiac arrest; TAE, transarterial catheter embolization; PPP, pre-peritoneal packing.

In total, 611 patients with pelvic fractures were included in this study. A flowchart of the inclusion process is shown in Figure 3.

Six patients with out-of-hospital cardiac arrest and two patients with missing data were excluded. Acute TAE was performed in 172 patients, and 14 patients were excluded because they had undergone PPP with TAE, which may have influenced the effects of TAE on hemostasis. Previously, PPP and TAE have been reported as “complementary procedures” performed to stop bleeding (23, 24). Although complementary (23, 25), PPP and TAE could be effective as a single or combined strategy, depending on the situation. In total, 158 (25.9%) patients met the inclusion criteria.

Table 2 summarizes the patients’ clinical characteristics. This study included 94 males (59.5%) and 64 females (40.5%), with a median age of 74 years. Eighty patients (50.6%) were transferred from other hospitals. The median injury severity score was 25. The proportion of patients with severe anatomic injuries with an abbreviated injury score ≥ 3 was the highest for the pelvis, followed by the chest, head, and abdomen (78.5, 34.2, 25.3, and 16.5%, respectively). The median systolic blood pressure on arrival was 110 mmHg, and the median GCS score was 14. The Tile Orthopaedic Trauma Association classifications and indication for TAE are presented in Table 2. There was an unknown fracture type in one patient because of the lack of a CT scan.

**Table 2:** General demographics of the patients with pelvic fracture who received acute angioembolization for pelvic injury.

Factors	No. (%) or Median (25, 75%) (*N* = 158)
Age (y/o)	74 (61, 81)
Sex	
Male	94 (59.5%)
Female	64 (40.5%)
Antiplatelet drug	26 (16.5%)
Anticoagulant drug	12 (7.6%)
Transfer, *n* (%)	80 (50.6%)
ISS (score)	25 (16, 34)
Severe anatomic injuries, *n* (%)	
Head AIS≧3	40 (25.3%)
Chest AIS≧3	54 (34.2%)
Abdomen AIS≧3	26 (16.5%)
Pelvis AIS≧3	124 (78.5%)
SBP upon ED arrival (mmHg)	110 (87, 132)
GCS upon ED arrival (total)	14 (13, 15)
Pelvic fracture type, *n* (%)	
Tile OTA classification	
A1	6 (3.8%)
A2	11 (7.0%)
A3	3 (1.9%)
B1	44 (27.9%) (including 5 associated acetabular fractures)
B2	26 (16.5%) (including 3 associated acetabular fractures)
B3	16 (10.1%) (including 1 associated acetabular fracture)
C1	23 (14.6%) (including 1 associated acetabular fracture)
C2	7 (4.4%)
C3	5 (3.1%)
Unknown	1 (0.6%)
Sacral fracture	2 (1.2%)
Acetabular fracture	14 (8.9%)
Indications for TAE	
1. Contrast extravasation on CT scan	134 (84.8%)
2. Massive hematoma on CT scan	13 (8.2%)
3. Unstable hemodynamics	11 (7.0%)
DT group, *n* (%)	45 (28.5%)
CT group, *n* (%)	113 (71.5%)

ISS, injury severity score; AIS, Abbreviated injury score; TAE, transcatheter arterial embolization; DT, door-to-TAE, SBP, systolic blood pressure; GCS, Glasgow coma scale; OTA, Orthopaedic Trauma Association; CT, Computed tomography; ED, Emergency department.

### 3.2 Relationship between mortality and decision-TAE time

**Figure 4:**
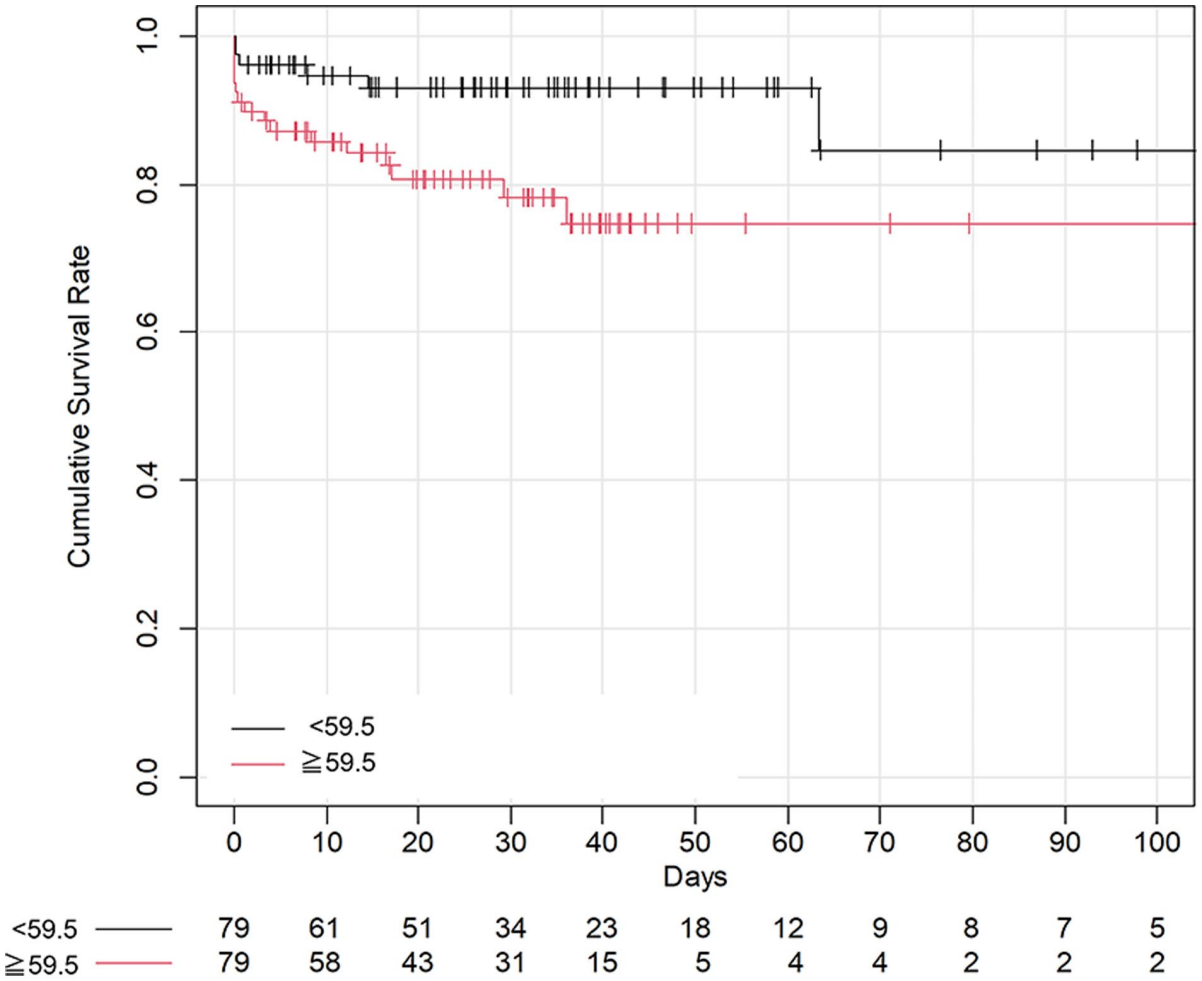
Kaplan–Meier curves of overall survival. The vertical axis shows the cumulative survival rate by Kaplan–Meier estimation. The horizontal axis shows the number of days since the baseline day. Marks in the curve indicate data censoring. The Kaplan–Meier curves of overall survival are compared, and a significant difference is observed between the patients above and below the median cutoff value for decision-TAE time (*p* = 0.02). TAE, transarterial catheter embolization.

The median decision-TAE time was 59.5 min (IQR: 40–87 min). Twenty-three patients died, and the mortality rate was 14.6%. Patients with decision-TAE time < 59.5 min had significantly higher survival rates than those with decision-TAE time ≥ 59.5 min (*p* = 0.02) as per the Kaplan–Meier curves (Figure 4). The hazard ratio was plotted when the reference was fixed at 105 min.

The multivariable Cox proportional hazards regression model adjusted for age, sex, GCS score, and transfer revealed that the longer the decision-TAE time, the higher the risk of mortality (Table 1). The optimism parameter was 0.168, indicating that the model was not overfitting. The results from the model with the CT/DT group added as a covariate also showed that the decision-TAE time was significant, thus achieving robustness of the results. After adjusting for age and sex, TAE duration was not significantly associated with mortality (Table 1). We also performed sensitivity analysis using Fine-Gray subdistribution hazard model with competing risk of death resulting from head trauma (Supplementary Table S2).

**Figure 5:**
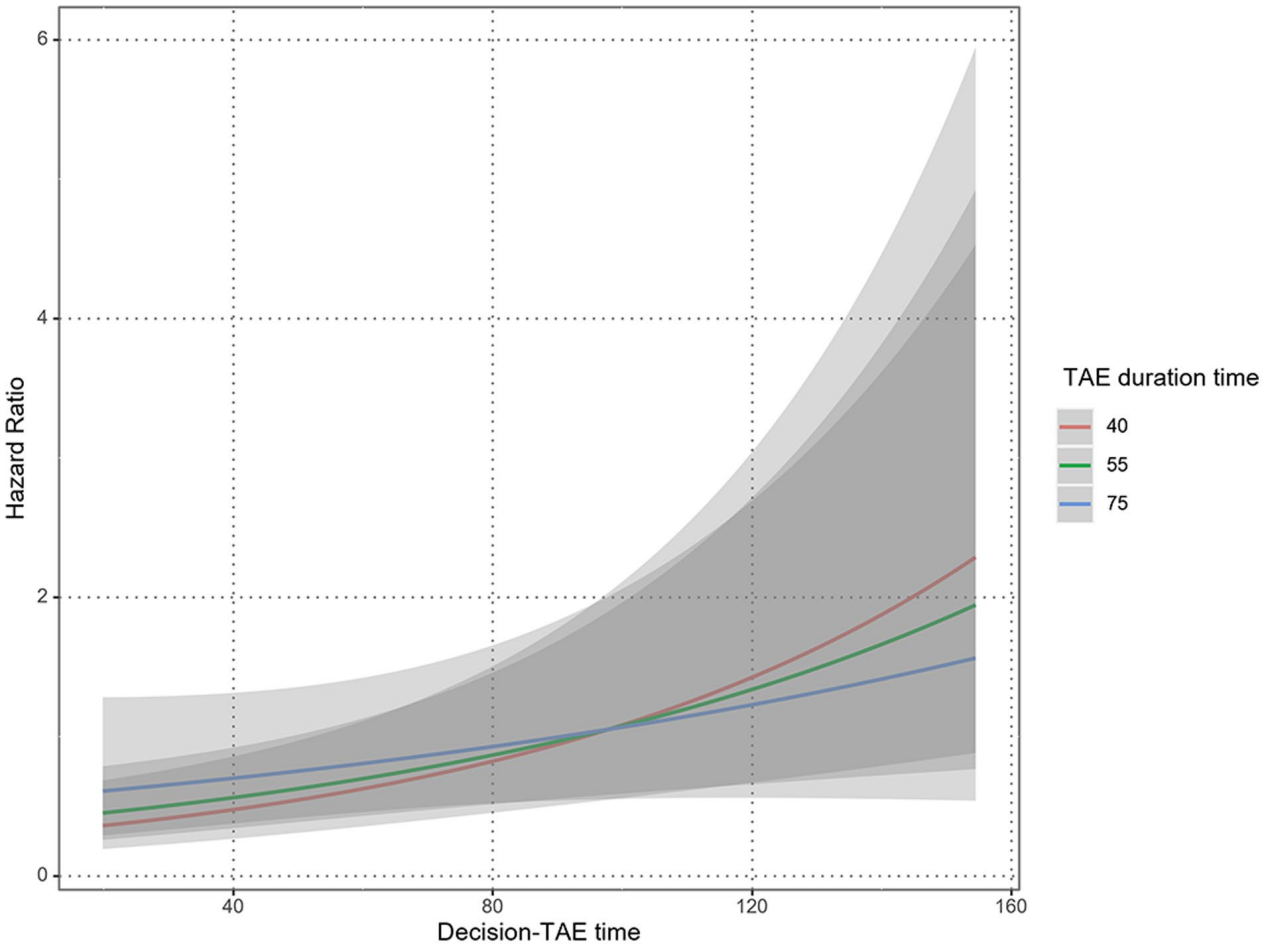
Interaction between TAE duration and decision-TAE time. Predicted plots of hazard ratios by Performed TAE time with median decision-TAE time as a reference are shown; the three solid lines correspond to the 25th, 50th, and 75th percentile of Performed TAE time, respectively. The gray shaded areas indicate 95% confidence intervals. Although the interaction between TAE duration and decision-TAE time is not significant (*p* = 0.109), TAE duration modified the effect of decision-TAE time on mortality. TAE, transarterial catheter embolization.

Although the interaction between TAE duration and decision-TAE time was not significant (*p* = 0.109), it indicated that TAE duration modified the effect of decision-TAE time on mortality (Figure 5).

### 3.3 Patient outcomes

The total number of arteries involved during TAE was 455, with a median value of three (IQR: 2–4). The locations of the arteries and embolic materials are summarized in Supplementary Tables S3, S4, respectively. There were 6 (3.8%) cases of REBOA. Two patients (1.3%) underwent secondary TAE for hemostasis. Sixty-eight patients (43.0%) underwent surgical management for pelvic fractures, including external fixation in 16 patients (10.1%) and internal fixation in 59 patients (37.3%). The median hospital length of stay was 26 (IQR: 11–41) days. The cause of death was unstable hemodynamics in three patients (1.9%), severe head trauma in nine patients (5.7%), unstable hemodynamics and severe head trauma in three patients (1.9%), and other causes including sepsis or respiratory failure in eight patients (5.1%). Patient outcomes are summarized in Table 3.

**Table 3:** Outcomes of the patients with pelvic fracture who received acute angioembolization for pelvic injury (*N* = 158).

Factors	No. (%) or Median (25, 75%)
Surgical management	68 (43.0%)
External fixation	16 (10.1%)
Internal fixation	59 (37.3%)
Combined REBOA	6 (3.8%)
Secondary TAE for hemostasis	2 (1.3%)
Mortality, *n* (%)	23 (14.6%)
Mean hospital length of stay (day)	26 (11, 41)
Reasons for death	
(1) Unstable hemodynamics, *n* (%)	3 (1.9%)
(2) Severe head trauma, *n* (%)	9 (5.7%)
(3) (1) and (2), *n* (%)	3 (1.9%)
(4) Other reasons, *n* (%)	8 (5.1%)

REBOA, resuscitative endovascular occlusion of the aorta; TAE, transarterial catheter embolization.

## 4 Discussion

The primary finding of this study was that long decision-TAE time resulted in a high risk of mortality. Sensitivity analysis with GCS replaced by RTS which both could represent the severity of patients based on the physiologic status, indicated that decision-TAE time was still significant. Moreover, although the actual TAE duration did not have a significant influence on decision-TAE time, the interaction between TAE duration and decision-TAE time was significant, indicating that TAE duration modified the effect of decision-TAE time on mortality.

Some reports have suggested the importance of early TAE for improving mortality (4, 15, 16, 26). In clinical settings, many variations exist in the circumstances surrounding patient delivery to the ED, and the patient’s condition upon delivery (27, 28), including the presence of associated injuries, severity of said injuries, and differences in vital signs (29). Moreover, they may have been transferred from another hospital and previously treated by prehospital medical professionals (30, 31). Physicians must decide upon a treatment plan for these patients, taking these factors into consideration (32). Hence, the actual effectiveness of shortening the delay from decision-making to actual TAE administration can be confirmed by analyzing the time from the decision to administer TAE to its administration and effect on the outcomes (29).

Reportedly, the time to angioembolization is longer than the time to PPP, partly owing to the higher availability of orthopedic surgeons compared with that of interventional radiologists (7, 12, 33); furthermore, TAE may be delayed at night or on weekends based on reports of other catheter-based interventions (16, 34, 35). In our institution, interventional radiologists and the equipment required for TAE are available at all times. Therefore, staff and equipment availability was not an issue in this study. The overall decision-TAE time was 60 min, even after performing other resuscitation procedures. Although PPP may have advantages over TAE, such as early start time (7, 12), most patients with pelvic fracture, even if unstable, can be managed with primary TAE strategies at centers with 24-h availability of interventional radiologists (36).

The effectiveness of REBOA for patients with unstable pelvic fractures has been reported (8, 9). In this study, there were seven (4.4%) cases of REBOA; however, there were no clear indications for REBOA in patients with pelvic fracture. Moreover, the consensus on REBOA indications, ideal patient populations, and outcomes is undecided, even among trauma specialties (37); therefore, further studies are needed. In our facility, we aim to complete TAE within 60 min, including treatment of other bleeding injuries. Our intervention analysis showed that TAE duration modified the effect of decision-TAE time on mortality, though the relationship between TAE duration and mortality was undetermined. Our results, as outlined in Figure 5, showed that patients with a decision-TAE time ≥ 105 min benefited from a long TAE duration, whereas patients with a decision-TAE time < 105 min benefitted from a short TAE duration. When the decision-TAE time increased from 105 min to 115 min, the risk increased by 1.15, 1.1, and 1.08 times for cases with TAE duration times of 40, 55, and 75 min, respectively. When the decision-TAE time was extended by an additional 10 min to 125 min, the risk increased by 1.3, 1.23, and 1.15 times, respectively. Conversely, when the decision-TAE time was reduced by 10 min from 105 min to 95 min, the risk was 0.88, 0.9, and 0.93 times, respectively. If the TAE time was reduced from 105 min to 20 min, the risk increased by 0.77, 0.81, and 0.86 times, respectively. This suggests that short decision-TAE and short procedure times might lead to improved mortality outcomes. To the best of our knowledge, this is the first study to discuss the relationship between TAE duration and mortality, as previous reports only speculated on this relationship.

We could not confirm the factors that influenced the decision-TAE time, except for hospital transfer; the expected parameters that could influence the severity of the patient’s condition, such as ISS, vital signs, and even associated injuries, were not related to decision-TAE time. Additionally, the transferred patients underwent TAE within a short duration, indicating that fast CT scanning can reduce decision-TAE time; hence, the development of fast imaging strategies is essential. Recent reports have suggested the effectiveness of hybrid emergency room systems (38, 39), hybrid operation rooms (40), and mobile angiography systems (41) for treating patients with trauma. These systems consist of an angiography-CT machine in a trauma resuscitation room and have the potential to provide new evidence in this field.

This study has some limitations. First, the performance of CT scan was dependent on the patient’s mode of admission; hence, we could not determine the severity of the patient’s condition based on the CT/DT stratification. The small number of CT/DT subgroups did not allow multivariable analysis. Caution may be warranted in univariable analysis results because the effects of confounding factors could not be excluded. Second, we could not clarify the actual durations of “decision time,” meaning that other decision-TAE times could be established, and if so, the results would change. Third, the results of this study cannot be generalized to other facilities that do not have the same interventional radiology coverage and equipment. Fourth, as the decision on treatment with REBOA was made by physicians, we could not analyze the impacts of REBOA in this study. Fifth, the time course of patients with pelvic injury varies according to their status; for some, there may be time to perform a CT scan before TAE because their vital signs are relatively stable, whereas for others, this may not be possible (42). Sixth, the impact of head injury or other injuries which could have a lethal impact on mortality, could not be separated. In patients with pelvic trauma, some patients with severe head injury were potentially included. Thus, the treatment strategy should be established based on overall injuries.

In conclusion, overall survival was significantly different between the patients above and below the median cutoff value for decision-TAE time, and the longer the decision-TAE time, the higher the risk of mortality. Our results suggest that decision-TAE time plays a key role in establishing resuscitation procedures in patients with pelvic fracture; thus, efforts to shorten the decision-TAE time are warranted.

## Data availability statement

The raw data supporting the conclusions of this article will be made available by the authors, without undue reservation.

## Ethics statement

The studies involving humans were approved by the medical ethics committee of Gifu University Graduate School of Medicine. The studies were conducted in accordance with the local legislation and institutional requirements. The ethics committee/institutional review board waived the requirement of written informed consent for participation from the participants or the participants’ legal guardians/next of kin because the need for informed consent from the patients was waived by the medical ethics committee of the institution because of the study’s retrospective nature. The manuscript presents research on animals that do not require ethical approval for their study.

## Author contributions

YM: Data curation, Writing – original draft. TM: Writing – original draft, Writing – review & editing. HO: Conceptualization, Supervision, Writing – review & editing. TI: Data curation, Methodology, Supervision, Writing – original draft. NK: Data curation, Writing – review & editing. MI: Data curation, Writing – review & editing. RK: Data curation, Writing – review & editing. TF: Data curation, Writing – review & editing. TY: Data curation, Writing – review & editing. SN: Data curation, Writing – review & editing. HK: Data curation, Writing – review & editing. MM: Conceptualization, Supervision, Writing – review & editing. SY: Conceptualization, Supervision, Writing – review & editing. SO: Conceptualization, Supervision, Writing – review & editing.

## Footnotes

^1^ https://www.r-project.org/
