# Supplementary material for: A short decision time for transcatheter embolization can better associate mortality in patients with pelvic fracture: a retrospective study
Source: Front Med (Lausanne). 2024 Jan 8;10:1329167. doi: 10.3389/fmed.2023.1329167 (PMC10800860; doi:10.3389/fmed.2023.1329167)
Supplement: Supplementary file 1 [file Table_1.docx]

**File name: Additional file 1**

**File type:** Word (.docx)

**Table S1.** Subgroup analysis by DT group and CT group were performed using univariate Cox regression models.

Table S1a: DT: Univariable Cox regression: Decision-TAE time

| Names | HR | 95%LCI | 95%UCI | p value |
| --- | --- | --- | --- | --- |
| Decision-TAE time | 1.002 | 0.975 | 1.029 | 0.908 |

Table S1b: DT: Univariable Cox regression: TAE duration time

| Names | HR | 95%LCI | 95%UCI | p value |
| --- | --- | --- | --- | --- |
| Decision-TAE time | 0.808 | 0.238 | 2.740 | 0.733 |

Table S1c: CT: Univariable Cox regression: Decision-TAE time

| Names | HR | 95%LCI | 95%UCI | p value |
| --- | --- | --- | --- | --- |
| Decision-TAE time | 1.007 | 1.000 | 1.013 | 0.046 |

Table S1d: CT: Univariable Cox regression: TAE duration time

| names | HR | 95%LCI | 95%UCI | p value |
| --- | --- | --- | --- | --- |
| Decision-TAE time | 0.882 | 0.443 | 1.753 | 0.720 |

**Table S2.** Sensitivity analysis by Fine–Gray subdistribution hazard model with competing risk of death resulting from head trauma.

**Multivariable Fine-Gray regression: Decision TAE time**

| names | HR | 95% LCI | 95% UCI | p value |
| --- | --- | --- | --- | --- |
| Decision-TAE time | 1.012 | 1.003 | 1.021 | 0.009 |
| GCS | 0.886 | 0.777 | 1.009 | 0.068 |
| Transfer | 1.025 | 0.339 | 3.099 | 0.970 |
| Age | 1.006 | 0.970 | 1.043 | 0.760 |
| Gender-female | 0.158 | 0.035 | 0.71 | 0.016 |

**Multivariable Fine-Gray regression: TAE duration time**

| names | HR | 95% LCI | 95% UCI | p value |
| --- | --- | --- | --- | --- |
| TAE duration time | 1.016 | 0.993 | 1.039 | 0.180 |
| Age | 1.007 | 0.972 | 1.043 | 0.710 |
| Gender-female | 0.181 | 0.037 | 0.879 | 0.034 |

Adjustment for GCS, transfer, age, and sex revealed that the longer the decision-TAE time, the higher the risk of mortality (HR: 1.012, 95%CI: 1.003–1.021, p=0.009). The TAE duration time was not significant (HR: 1.016, 95% CI: 0.993–1.039, p=0.18).

TAE, transcatheter arterial embolization; GCS, Glasgow coma scale; LCI, lower confidence interval; UCI, upper confidence interval; HR: hazard ratio

**Table S3.** Summary of the number of arteries on which TAE was performed, localizations of TAE, and number of patients who needed embolizations out of the pelvis.

| Number and localizations of embolizations | N |  | |
| --- | --- | --- | --- |
|  |  | |  |
| Number of embolizations, Median (25%, 75%) | 158 | | 3 (2, 4) |
|  |  | |  |
| Localizations of embolizations No. (%) | 455 | |  |
| Internal Iliac artery |  | | 81 (17.8%) |
| External Iliac artery |  | | 5 (1.1%) |
| Superior gluteal artery |  | | 56 (12.3%) |
| Inferior gluteal artery |  | | 24 (5.3%) |
| Sacral artery＊ |  | | 59 (13.0%) |
| Obturator artery |  | | 57 (12.5%) |
| Iliolumbar artery |  | | 51 (11.2%) |
| Lumbar artery |  | | 28 (6.2%) |
| Internal pudendal artery |  | | 25 (5.5%) |
| Branches of femoral artery |  | | 15 (3.3%) |
| Inferior epigastric artery |  | | 13 (2.9%) |
| Deep iliac circumflex artery |  | | 7 (1.5%) |
| Other arteries in pelvis |  | | 3 (0.7%) |
| Other arteries out of pelvis |  | | 31 (6.8%) |
|  |  | |  |
| Number of patients who needed embolizations out of pelvis No. (%) | 158 | | 20 (12.7%) |
|  |  | |  |
| ＊Sacral artery includes median sacral arteries or lateral sacral arteries.  TAE, transcatheter arterial embolization | | |  |

**Table S4.** Summary of embolic materials utilized in TAE.

| Embolic Materials No. (%) | |  |  |
| --- | --- | --- | --- |
| Gelatin(G) | NBCA(N) | Coiling(C) | N=158 |
| **+** | **-** | **-** | 133 (84.2%) |
| **+** | **+** | **-** | 2 (1.3%) |
| **+** | **-** | **+** | 19 (12.0%) |
| **+** | **+** | **+** | 1 (0.6%) |
| **-** | **+** | **-** | 2 (1.3%) |
| **-** | **-** | **+** | 1 (0.6%) |
|  |  |  |  |
| NBCA: n-butyl-2-cyanoacrylate | | |  |
